# Bis(1-methyl-1*H*-imidazole-κ*N* ^3^)bis­[2-(naphthalen-1-yl)acetato-κ*O*]copper(II) monohydrate

**DOI:** 10.1107/S1600536811049439

**Published:** 2011-11-25

**Authors:** Fu-Jun Yin, Li-Jun Han, Shu-Ping Yang, Xing-You Xu, Yu Gu

**Affiliations:** a Jiangsu Marine Resources Development, Research Institute, Huaihai Institute of Technology, Lianyungang 222005, People’s Republic of China; b Department of Mathematics and Science, Huaihai Institute of Technology, Lianyungang 222005, People’s Republic of China; c Department of Chemical Engineering, Huaihai Institute of Technology, Lianyungang 222005, People’s Republic of China; d Department of Chemical Engineering, Huaiyin Insititute of Technology, Huaiyin 223003, People’s Republic of China; e Qian’an College, Hebei United University, Tangshan 063009, People’s Republic of China

## Abstract

In the crystal structure of the title compound, [Cu(C_12_H_9_O_2_)_2_(C_4_H_6_N_2_)_2_]·H_2_O, the Cu^II^ atom is coordinated by two 2-(naphthalen-1-yl)acetate anions and two 1-methyl­imidazole ligands, giving monomeric complexes with a square-planar coordination environment. Two complex mol­ecules and two water mol­ecules form a centrosymmetric ring system *via* O—H⋯O hydrogen bonds.

## Related literature

For the pharmacological potential of metal complexes with imidazole, see: Boiani & Gonzales (2005 ▶); Parshina & Trofimov (2011 ▶). For the coordination chemistry of 1-naphthyl­acetate ligands, see: Yin *et al.* (2010 ▶); Chen *et al.* (2004 ▶); Yang *et al.* (2008 ▶); Tang *et al.* (2006 ▶); Ji *et al.* (2011 ▶).
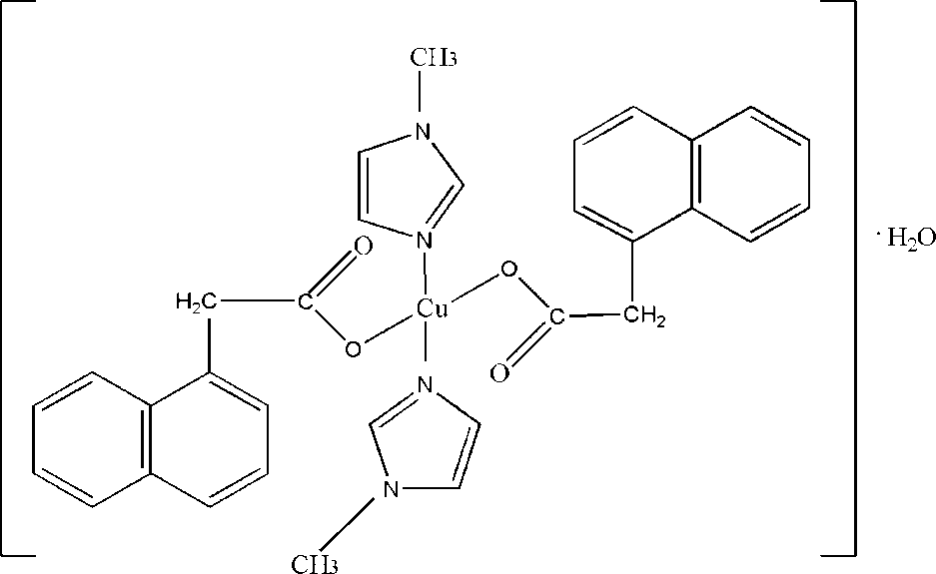


## Experimental

### 

#### Crystal data

[Cu(C_12_H_9_O_2_)_2_(C_4_H_6_N_2_)_2_]·H_2_O
*M* *_r_* = 616.16
Triclinic, 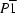

*a* = 8.7213 (10) Å
*b* = 12.8689 (14) Å
*c* = 13.5787 (15) Å
α = 107.223 (1)°
β = 90.295 (2)°
γ = 90.931 (1)°
*V* = 1455.4 (3) Å^3^
*Z* = 2
Mo *K*α radiation
μ = 0.80 mm^−1^
*T* = 298 K
0.20 × 0.20 × 0.20 mm

#### Data collection

Bruker APEXII CCD area-detector diffractometer
Absorption correction: multi-scan (*SADABS*; Sheldrick, 1996 ▶) *T*_min_ = 0.843, *T*_max_ = 0.843
11175 measured reflections
5094 independent reflections
3534 reflections with *I* > 2σ(*I*)
*R*_int_ = 0.040

#### Refinement

*R*[*F* ^2^ > 2σ(*F* ^2^)] = 0.045
*wR*(*F* ^2^) = 0.102
*S* = 1.03
5094 reflections
381 parameters
H-atom parameters constrained
Δρ_max_ = 0.26 e Å^−3^
Δρ_min_ = −0.31 e Å^−3^

### 

Data collection: *APEX2* (Bruker 2007 ▶); cell refinement: *SAINT* (Bruker 2007 ▶); data reduction: *SAINT*; program(s) used to solve structure: *SHELXS97* (Sheldrick, 2008 ▶); program(s) used to refine structure: *SHELXL97* (Sheldrick, 2008 ▶); molecular graphics: *SHELXTL* (Sheldrick, 2008 ▶); software used to prepare material for publication: *SHELXTL*.

## Supplementary Material

Crystal structure: contains datablock(s) I, global. DOI: 10.1107/S1600536811049439/im2340sup1.cif

Structure factors: contains datablock(s) I. DOI: 10.1107/S1600536811049439/im2340Isup2.hkl

Additional supplementary materials:  crystallographic information; 3D view; checkCIF report

## Figures and Tables

**Table 1:** Hydrogen-bond geometry (Å, °)

*D*—H⋯*A*	*D*—H	H⋯*A*	*D*⋯*A*	*D*—H⋯*A*
O1*W*—H1*WA*⋯O1	0.85	1.97	2.789 (3)	163
O1*W*—H1*WB*⋯O3^i^	0.85	2.07	2.904 (3)	167

Symmetry code: (i) 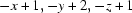.

**Fig. 1.**
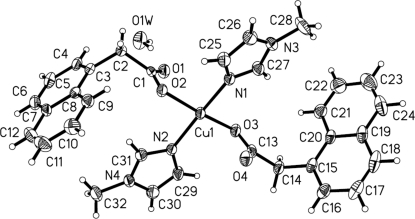
Crystal structure of the title compound with displacement ellipsoids drawn at the 30% probability level.

**Fig. 2.**
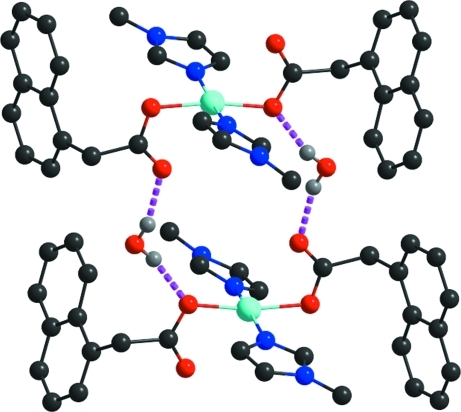
Centrosymmetric ring system of the title compound including lattice water molecules. Hydrogen bonds are shown as dashed lines, and H atoms not involved in hydrogen bonding are omitted for clarity.

## supplementary crystallographic information

### Comment

Self-assembly of supramolecular architectures based on naphthalene-1-yl-acetate ligands has attracted much attention during recent decades (Yin *et al.*, 2010; Chen *et al.*, 2004; Yang *et al.*, 2008; Tang *et al.*,2006; Ji *et al.*, 2011). Recent studies reveal that metal complexes with substituted imidazole and carboxylate ligands are interesting for  medicinal chemists who explore their various pharmacological potentials (Boiani & Gonzales, 2005; Parshina & Trofimov, 2011). The crystal structure of the title compound was determined as part of an ongoing study on the properties of copper complexes containing imidazole ligands.

In the crystal structure of the title compound [Cu(C_12_H_9_O_2_)_2_(C_4_H_6_N_2_)_2_]H_2_O, each copper cation is coordinated by two N atoms of different 1-methylimidazole ligands and by two carboxyl O atoms of distinct naphthalene-1-yl-acetate anions within a square planar coordination sphere (Fig. 1). The Cu—N and Cu—O bond lengths are 1.985 (3) and 1.974 (2) Å, respectively. The asymmetric unit consits of one Cu^II^ cation, two neutral imidazole ligands, two anionic carboxylate ligands and one lattic water in general positions. It is noteworthy that there exist strong hydrogen-bonding interactions (Table 1, Fig.2) involving the carboxy group oxygen atoms of 1-naphthylacetate ligands as well as  water molecules. The molecules fomr centrosymmetric ring systems by O—H···O hydrogen bonds

### Experimental

The title compound was synthesized by the reaction of Cu(NO_3_)_2_ × 3 H_2_O (72.3 mg, 0.3 mmol), naphthalene-1-yl-acetic acid (93 mg, 0.5 mmol), 1-methylimidazole (32.8 mg, 0.4 mmol) and NaOH (20 mg, 0.5 mmol) in 6 mL of a water-ethanol (2:1) mixture under solvothermal conditions. The mixture was homogenized and transferred into a sealed Teflon-lined solvothermal bomb (volume: 25 ml) and heated to 140°C for three days. After cooling green crystals of the title compound were obtained, which were washed with distilled water and absolute ethanol (yield: 38.7% based on Cu(NO_3_)_2_ × 3 H_2_O ).

### Refinement

H atoms were placed in calculated positions, with C—H = 0.93 or 0.97 Å and included in the final cycles of refinement using a riding model with U_iso_(H) = 1.2U_eq_(parent atom).Water H atoms were located in Fourier difference maps and refined isotropically.

### Crystal data

**Table:**

[Cu(C_12_H_9_O_2_)_2_(C_4_H_6_N_2_)_2_]·H_2_O	*Z* = 2
*M**_r_* = 616.16	*F*(000) = 642
Triclinic, *P*1	*D*_x_ = 1.406 Mg m^−^^3^
Hall symbol: -P 1	Mo *K*α radiation, λ = 0.71073 Å
*a* = 8.7213 (10) Å	Cell parameters from 1644 reflections
*b* = 12.8689 (14) Å	θ = 2.3–19.2°
*c* = 13.5787 (15) Å	µ = 0.80 mm^−^^1^
α = 107.223 (1)°	*T* = 298 K
β = 90.295 (2)°	Prism, green
γ = 90.931 (1)°	0.20 × 0.20 × 0.20 mm
*V* = 1455.4 (3) Å^3^	

### Data collection

**Table:**

Bruker APEXII CCD area-detector diffractometer	5094 independent reflections
Radiation source: fine-focus sealed tube	3534 reflections with *I* > 2σ(*I*)
graphite	*R*_int_ = 0.040
φ and ω scans	θ_max_ = 25.0°, θ_min_ = 2.3°
Absorption correction: multi-scan (*SADABS*; Sheldrick, 1996)	*h* = −10→10
*T*_min_ = 0.843, *T*_max_ = 0.843	*k* = −15→15
11175 measured reflections	*l* = −15→16

### Refinement

**Table:**

Refinement on *F*^2^	Primary atom site location: structure-invariant direct methods
Least-squares matrix: full	Secondary atom site location: difference Fourier map
*R*[*F*^2^ > 2σ(*F*^2^)] = 0.045	Hydrogen site location: inferred from neighbouring sites
*wR*(*F*^2^) = 0.102	H-atom parameters constrained
*S* = 1.03	*w* = 1/[σ^2^(*F*_o_^2^) + (0.0371*P*)^2^ + 0.1784*P*] where *P* = (*F*_o_^2^ + 2*F*_c_^2^)/3
5094 reflections	(Δ/σ)_max_ = 0.001
381 parameters	Δρ_max_ = 0.26 e Å^−^^3^
0 restraints	Δρ_min_ = −0.31 e Å^−^^3^

### Special details

**Table:**

Geometry. All esds (except the esd in the dihedral angle between two l.s. planes) are estimated using the full covariance matrix. The cell esds are taken into account individually in the estimation of esds in distances, angles and torsion angles; correlations between esds in cell parameters are only used when they are defined by crystal symmetry. An approximate (isotropic) treatment of cell esds is used for estimating esds involving l.s. planes.
Refinement. Refinement of F^2^ against ALL reflections. The weighted R-factor wR and goodness of fit S are based on F^2^, conventional R-factors R are based on F, with F set to zero for negative F^2^. The threshold expression of F^2^ > 2sigma(F^2^) is used only for calculating R-factors(gt) etc. and is not relevant to the choice of reflections for refinement. R-factors based on F^2^ are statistically about twice as large as those based on F, and R- factors based on ALL data will be even larger.

### Fractional atomic coordinates and isotropic or equivalent isotropic displacement parameters (Å^2^)

**Table:**

	*x*	*y*	*z*	*U*_iso_*/*U*_eq_	
C1	0.7153 (4)	0.7870 (3)	0.6104 (3)	0.0395 (8)	
C2	0.7391 (4)	0.6657 (2)	0.5639 (3)	0.0490 (9)	
H2A	0.6505	0.6333	0.5219	0.059*	
H2B	0.7486	0.6320	0.6187	0.059*	
C3	0.8829 (4)	0.6438 (2)	0.4976 (3)	0.0444 (8)	
C4	0.8703 (4)	0.6226 (3)	0.3940 (3)	0.0500 (9)	
H4	0.7735	0.6199	0.3643	0.060*	
C5	0.9998 (5)	0.6045 (3)	0.3301 (3)	0.0630 (11)	
H5	0.9880	0.5891	0.2590	0.076*	
C6	1.1423 (5)	0.6098 (3)	0.3728 (3)	0.0587 (10)	
H6	1.2279	0.5991	0.3307	0.070*	
C7	1.1612 (4)	0.6314 (3)	0.4797 (3)	0.0470 (9)	
C8	1.0292 (4)	0.6489 (2)	0.5452 (3)	0.0435 (8)	
C9	1.0541 (4)	0.6709 (3)	0.6524 (3)	0.0479 (9)	
H9	0.9703	0.6810	0.6960	0.058*	
C10	1.1973 (5)	0.6777 (3)	0.6928 (3)	0.0639 (11)	
H10	1.2109	0.6938	0.7637	0.077*	
C11	1.3237 (5)	0.6609 (3)	0.6291 (4)	0.0746 (13)	
H11	1.4216	0.6652	0.6577	0.090*	
C12	1.3065 (4)	0.6384 (3)	0.5264 (4)	0.0653 (11)	
H12	1.3932	0.6273	0.4852	0.078*	
C13	0.7022 (4)	1.1979 (3)	0.8398 (3)	0.0435 (8)	
C14	0.6624 (4)	1.3180 (2)	0.8668 (2)	0.0486 (9)	
H14A	0.7555	1.3604	0.8673	0.058*	
H14B	0.5946	1.3286	0.8139	0.058*	
C15	0.5856 (4)	1.3596 (3)	0.9707 (3)	0.0461 (9)	
C16	0.6705 (5)	1.4139 (3)	1.0546 (3)	0.0587 (10)	
H16	0.7744	1.4269	1.0472	0.070*	
C17	0.6043 (6)	1.4509 (3)	1.1527 (3)	0.0729 (13)	
H17	0.6643	1.4883	1.2093	0.088*	
C18	0.4542 (6)	1.4321 (3)	1.1650 (3)	0.0736 (13)	
H18	0.4120	1.4561	1.2304	0.088*	
C19	0.3595 (5)	1.3768 (3)	1.0802 (3)	0.0572 (10)	
C20	0.4261 (4)	1.3412 (3)	0.9806 (3)	0.0448 (9)	
C21	0.3277 (5)	1.2891 (3)	0.8967 (3)	0.0597 (10)	
H21	0.3683	1.2649	0.8308	0.072*	
C22	0.1773 (5)	1.2735 (4)	0.9093 (4)	0.0807 (13)	
H22	0.1156	1.2400	0.8524	0.097*	
C23	0.1130 (6)	1.3074 (4)	1.0074 (5)	0.0928 (16)	
H23	0.0093	1.2948	1.0157	0.111*	
C24	0.2010 (6)	1.3585 (4)	1.0903 (4)	0.0837 (15)	
H24	0.1565	1.3820	1.1551	0.100*	
C25	0.5448 (4)	0.8706 (3)	0.8592 (3)	0.0524 (9)	
H25	0.6224	0.8212	0.8566	0.063*	
C26	0.4117 (4)	0.8717 (3)	0.9070 (3)	0.0579 (10)	
H26	0.3797	0.8238	0.9427	0.069*	
C27	0.4195 (4)	1.0032 (3)	0.8372 (3)	0.0518 (9)	
H27	0.3913	1.0633	0.8169	0.062*	
C28	0.1776 (4)	0.9878 (4)	0.9308 (3)	0.0884 (15)	
H28A	0.1519	1.0545	0.9172	0.133*	
H28B	0.1746	0.9981	1.0037	0.133*	
H28C	0.1050	0.9318	0.8964	0.133*	
C29	0.9660 (4)	1.1131 (3)	0.6535 (3)	0.0530 (10)	
H29	0.9081	1.1756	0.6656	0.064*	
C30	1.1062 (4)	1.1006 (3)	0.6125 (3)	0.0574 (10)	
H30	1.1621	1.1517	0.5906	0.069*	
C31	1.0373 (4)	0.9532 (3)	0.6472 (3)	0.0486 (9)	
H31	1.0398	0.8832	0.6536	0.058*	
C32	1.2957 (4)	0.9490 (3)	0.5669 (3)	0.0702 (12)	
H32A	1.2955	0.8747	0.5679	0.105*	
H32B	1.3799	0.9881	0.6081	0.105*	
H32C	1.3064	0.9513	0.4973	0.105*	
Cu1	0.72561 (4)	0.98739 (3)	0.73588 (3)	0.03873 (15)	
N1	0.5513 (3)	0.9531 (2)	0.8147 (2)	0.0434 (7)	
N2	0.9208 (3)	1.0192 (2)	0.67509 (19)	0.0403 (7)	
N3	0.3314 (3)	0.9560 (2)	0.8930 (2)	0.0527 (8)	
N4	1.1514 (3)	0.9992 (2)	0.6089 (2)	0.0461 (7)	
O1	0.6406 (3)	0.83829 (19)	0.56361 (17)	0.0543 (6)	
O2	0.7793 (2)	0.83265 (16)	0.69796 (17)	0.0433 (6)	
O3	0.6513 (2)	1.13568 (16)	0.75272 (17)	0.0444 (6)	
O4	0.7809 (3)	1.16518 (18)	0.89921 (18)	0.0540 (6)	
O1W	0.5269 (3)	0.7509 (2)	0.36319 (19)	0.0803 (9)	
H1WA	0.5433	0.7839	0.4266	0.120*	
H1WB	0.4770	0.7933	0.3378	0.120*	

### Atomic displacement parameters (Å^2^)

**Table:**

	*U*^11^	*U*^22^	*U*^33^	*U*^12^	*U*^13^	*U*^23^
C1	0.0288 (18)	0.0333 (19)	0.053 (2)	0.0010 (15)	0.0151 (17)	0.0078 (17)
C2	0.0364 (19)	0.0341 (19)	0.068 (2)	0.0010 (16)	0.0061 (17)	0.0025 (17)
C3	0.043 (2)	0.0273 (18)	0.057 (2)	0.0033 (15)	0.0049 (18)	0.0016 (16)
C4	0.050 (2)	0.0354 (19)	0.053 (2)	0.0078 (17)	−0.0026 (18)	−0.0050 (17)
C5	0.082 (3)	0.046 (2)	0.054 (2)	0.016 (2)	0.010 (2)	0.0012 (19)
C6	0.057 (3)	0.047 (2)	0.072 (3)	0.0129 (19)	0.023 (2)	0.016 (2)
C7	0.039 (2)	0.0324 (19)	0.072 (3)	0.0081 (16)	0.0143 (19)	0.0175 (18)
C8	0.040 (2)	0.0250 (17)	0.066 (3)	0.0041 (15)	0.0048 (18)	0.0145 (17)
C9	0.048 (2)	0.041 (2)	0.057 (2)	0.0063 (17)	0.0068 (18)	0.0174 (18)
C10	0.061 (3)	0.064 (3)	0.073 (3)	0.005 (2)	−0.011 (2)	0.029 (2)
C11	0.045 (3)	0.081 (3)	0.109 (4)	0.003 (2)	−0.009 (3)	0.046 (3)
C12	0.040 (2)	0.071 (3)	0.094 (3)	0.011 (2)	0.013 (2)	0.037 (3)
C13	0.049 (2)	0.038 (2)	0.045 (2)	0.0085 (17)	0.0225 (18)	0.0130 (18)
C14	0.060 (2)	0.0341 (19)	0.050 (2)	0.0078 (17)	0.0128 (18)	0.0092 (17)
C15	0.065 (3)	0.0293 (18)	0.044 (2)	0.0163 (18)	0.0044 (19)	0.0098 (17)
C16	0.069 (3)	0.039 (2)	0.063 (3)	0.0108 (19)	−0.006 (2)	0.007 (2)
C17	0.110 (4)	0.048 (2)	0.051 (3)	0.023 (3)	−0.017 (3)	−0.001 (2)
C18	0.125 (4)	0.054 (3)	0.043 (3)	0.044 (3)	0.017 (3)	0.014 (2)
C19	0.077 (3)	0.047 (2)	0.052 (3)	0.029 (2)	0.019 (2)	0.020 (2)
C20	0.058 (2)	0.0325 (19)	0.044 (2)	0.0147 (17)	0.0094 (18)	0.0109 (16)
C21	0.062 (3)	0.049 (2)	0.065 (3)	0.009 (2)	0.000 (2)	0.010 (2)
C22	0.063 (3)	0.074 (3)	0.102 (4)	0.007 (3)	−0.009 (3)	0.022 (3)
C23	0.061 (3)	0.092 (4)	0.135 (5)	0.022 (3)	0.020 (3)	0.047 (4)
C24	0.088 (4)	0.081 (3)	0.095 (4)	0.040 (3)	0.049 (3)	0.043 (3)
C25	0.060 (2)	0.048 (2)	0.055 (2)	0.0086 (19)	0.0147 (19)	0.0227 (19)
C26	0.071 (3)	0.055 (2)	0.052 (2)	−0.001 (2)	0.015 (2)	0.022 (2)
C27	0.054 (2)	0.045 (2)	0.061 (2)	0.0096 (19)	0.0161 (19)	0.0212 (19)
C28	0.052 (3)	0.114 (4)	0.106 (4)	0.012 (3)	0.039 (3)	0.042 (3)
C29	0.061 (2)	0.035 (2)	0.066 (3)	0.0124 (18)	0.022 (2)	0.0186 (19)
C30	0.062 (3)	0.047 (2)	0.065 (3)	−0.001 (2)	0.017 (2)	0.021 (2)
C31	0.050 (2)	0.038 (2)	0.059 (2)	0.0021 (18)	0.0131 (19)	0.0160 (18)
C32	0.047 (2)	0.068 (3)	0.097 (3)	0.014 (2)	0.023 (2)	0.025 (2)
Cu1	0.0421 (3)	0.0321 (2)	0.0417 (3)	0.00717 (17)	0.00974 (18)	0.00997 (18)
N1	0.0499 (18)	0.0345 (15)	0.0460 (17)	0.0054 (14)	0.0122 (14)	0.0116 (13)
N2	0.0433 (17)	0.0325 (15)	0.0437 (17)	0.0034 (13)	0.0083 (13)	0.0089 (13)
N3	0.0475 (18)	0.0539 (19)	0.0560 (19)	0.0007 (16)	0.0189 (15)	0.0150 (16)
N4	0.0430 (17)	0.0433 (17)	0.0508 (18)	0.0046 (14)	0.0133 (14)	0.0118 (14)
O1	0.0524 (15)	0.0514 (15)	0.0562 (16)	0.0112 (12)	0.0009 (12)	0.0111 (13)
O2	0.0456 (14)	0.0348 (13)	0.0479 (15)	0.0066 (11)	0.0114 (11)	0.0092 (11)
O3	0.0512 (14)	0.0351 (13)	0.0468 (15)	0.0091 (11)	0.0119 (11)	0.0113 (11)
O4	0.0604 (16)	0.0506 (15)	0.0530 (16)	0.0123 (13)	0.0076 (13)	0.0177 (13)
O1W	0.091 (2)	0.085 (2)	0.0606 (18)	0.0234 (17)	−0.0078 (15)	0.0132 (15)

### Geometric parameters (Å, °)

**Table:**

C1—O1	1.233 (4)	C19—C20	1.423 (5)
C1—O2	1.280 (4)	C20—C21	1.416 (5)
C1—C2	1.519 (4)	C21—C22	1.343 (5)
C2—C3	1.528 (4)	C21—H21	0.9300
C2—H2A	0.9700	C22—C23	1.396 (6)
C2—H2B	0.9700	C22—H22	0.9300
C3—C4	1.355 (4)	C23—C24	1.351 (6)
C3—C8	1.419 (4)	C23—H23	0.9300
C4—C5	1.408 (5)	C24—H24	0.9300
C4—H4	0.9300	C25—C26	1.331 (4)
C5—C6	1.360 (5)	C25—N1	1.369 (4)
C5—H5	0.9300	C25—H25	0.9300
C6—C7	1.403 (5)	C26—N3	1.360 (4)
C6—H6	0.9300	C26—H26	0.9300
C7—C12	1.404 (5)	C27—N1	1.319 (4)
C7—C8	1.438 (4)	C27—N3	1.341 (4)
C8—C9	1.413 (4)	C27—H27	0.9300
C9—C10	1.353 (4)	C28—N3	1.460 (4)
C9—H9	0.9300	C28—H28A	0.9600
C10—C11	1.385 (5)	C28—H28B	0.9600
C10—H10	0.9300	C28—H28C	0.9600
C11—C12	1.345 (5)	C29—C30	1.338 (4)
C11—H11	0.9300	C29—N2	1.377 (4)
C12—H12	0.9300	C29—H29	0.9300
C13—O4	1.226 (4)	C30—N4	1.356 (4)
C13—O3	1.288 (4)	C30—H30	0.9300
C13—C14	1.525 (4)	C31—N2	1.319 (4)
C14—C15	1.517 (4)	C31—N4	1.334 (4)
C14—H14A	0.9700	C31—H31	0.9300
C14—H14B	0.9700	C32—N4	1.463 (4)
C15—C16	1.356 (5)	C32—H32A	0.9600
C15—C20	1.421 (5)	C32—H32B	0.9600
C16—C17	1.406 (5)	C32—H32C	0.9600
C16—H16	0.9300	Cu1—O2	1.969 (2)
C17—C18	1.348 (6)	Cu1—O3	1.974 (2)
C17—H17	0.9300	Cu1—N1	1.981 (3)
C18—C19	1.415 (5)	Cu1—N2	1.985 (3)
C18—H18	0.9300	O1W—H1WA	0.8500
C19—C24	1.413 (6)	O1W—H1WB	0.8501
			
O1—C1—O2	122.5 (3)	C15—C20—C19	118.9 (3)
O1—C1—C2	120.6 (3)	C22—C21—C20	122.0 (4)
O2—C1—C2	116.9 (3)	C22—C21—H21	119.0
C1—C2—C3	111.2 (3)	C20—C21—H21	119.0
C1—C2—H2A	109.4	C21—C22—C23	120.5 (5)
C3—C2—H2A	109.4	C21—C22—H22	119.8
C1—C2—H2B	109.4	C23—C22—H22	119.8
C3—C2—H2B	109.4	C24—C23—C22	120.3 (5)
H2A—C2—H2B	108.0	C24—C23—H23	119.9
C4—C3—C8	120.3 (3)	C22—C23—H23	119.9
C4—C3—C2	119.8 (3)	C23—C24—C19	121.0 (4)
C8—C3—C2	119.8 (3)	C23—C24—H24	119.5
C3—C4—C5	121.9 (3)	C19—C24—H24	119.5
C3—C4—H4	119.1	C26—C25—N1	110.2 (3)
C5—C4—H4	119.1	C26—C25—H25	125.0
C6—C5—C4	119.6 (4)	N1—C25—H25	124.8
C6—C5—H5	120.2	C25—C26—N3	106.7 (3)
C4—C5—H5	120.2	C25—C26—H26	126.6
C5—C6—C7	120.7 (4)	N3—C26—H26	126.7
C5—C6—H6	119.7	N1—C27—N3	111.1 (3)
C7—C6—H6	119.7	N1—C27—H27	124.5
C6—C7—C12	122.1 (3)	N3—C27—H27	124.4
C6—C7—C8	119.9 (3)	N3—C28—H28A	109.5
C12—C7—C8	118.0 (4)	N3—C28—H28B	109.5
C9—C8—C3	124.6 (3)	H28A—C28—H28B	109.5
C9—C8—C7	117.8 (3)	N3—C28—H28C	109.5
C3—C8—C7	117.6 (3)	H28A—C28—H28C	109.5
C10—C9—C8	121.4 (3)	H28B—C28—H28C	109.5
C10—C9—H9	119.3	C30—C29—N2	109.5 (3)
C8—C9—H9	119.3	C30—C29—H29	125.2
C9—C10—C11	120.3 (4)	N2—C29—H29	125.2
C9—C10—H10	119.8	C29—C30—N4	107.0 (3)
C11—C10—H10	119.8	C29—C30—H30	126.6
C12—C11—C10	120.8 (4)	N4—C30—H30	126.4
C12—C11—H11	119.6	N2—C31—N4	111.6 (3)
C10—C11—H11	119.6	N2—C31—H31	124.2
C11—C12—C7	121.7 (4)	N4—C31—H31	124.1
C11—C12—H12	119.1	N4—C32—H32A	109.5
C7—C12—H12	119.1	N4—C32—H32B	109.5
O4—C13—O3	123.2 (3)	H32A—C32—H32B	109.5
O4—C13—C14	120.0 (3)	N4—C32—H32C	109.5
O3—C13—C14	116.7 (3)	H32A—C32—H32C	109.5
C15—C14—C13	112.8 (3)	H32B—C32—H32C	109.5
C15—C14—H14A	109.0	O2—Cu1—O3	170.52 (10)
C13—C14—H14A	109.0	O2—Cu1—N1	88.15 (10)
C15—C14—H14B	109.0	O3—Cu1—N1	92.02 (10)
C13—C14—H14B	109.0	O2—Cu1—N2	89.41 (9)
H14A—C14—H14B	107.8	O3—Cu1—N2	91.82 (9)
C16—C15—C20	120.1 (3)	N1—Cu1—N2	170.95 (11)
C16—C15—C14	119.5 (3)	C27—N1—C25	105.1 (3)
C20—C15—C14	120.5 (3)	C27—N1—Cu1	128.9 (2)
C15—C16—C17	121.1 (4)	C25—N1—Cu1	126.0 (2)
C15—C16—H16	119.4	C31—N2—C29	104.9 (3)
C17—C16—H16	119.4	C31—N2—Cu1	126.5 (2)
C18—C17—C16	120.2 (4)	C29—N2—Cu1	128.7 (2)
C18—C17—H17	119.9	C27—N3—C26	107.0 (3)
C16—C17—H17	119.9	C27—N3—C28	126.8 (3)
C17—C18—C19	121.2 (4)	C26—N3—C28	126.2 (3)
C17—C18—H18	119.4	C31—N4—C30	107.0 (3)
C19—C18—H18	119.4	C31—N4—C32	126.9 (3)
C24—C19—C18	122.4 (4)	C30—N4—C32	126.1 (3)
C24—C19—C20	119.1 (4)	C1—O2—Cu1	106.53 (19)
C18—C19—C20	118.5 (4)	C13—O3—Cu1	108.00 (19)
C21—C20—C15	123.9 (3)	H1WA—O1W—H1WB	107.7
C21—C20—C19	117.3 (4)		
			
O1—C1—C2—C3	89.1 (4)	C20—C21—C22—C23	−0.9 (6)
O2—C1—C2—C3	−89.1 (4)	C21—C22—C23—C24	1.5 (7)
C1—C2—C3—C4	−97.6 (4)	C22—C23—C24—C19	−1.2 (7)
C1—C2—C3—C8	80.5 (4)	C18—C19—C24—C23	178.5 (4)
C8—C3—C4—C5	0.3 (5)	C20—C19—C24—C23	0.4 (6)
C2—C3—C4—C5	178.4 (3)	N1—C25—C26—N3	0.1 (4)
C3—C4—C5—C6	−1.0 (5)	N2—C29—C30—N4	−0.6 (4)
C4—C5—C6—C7	1.0 (5)	N3—C27—N1—C25	−0.2 (4)
C5—C6—C7—C12	−179.4 (3)	N3—C27—N1—Cu1	−179.4 (2)
C5—C6—C7—C8	−0.4 (5)	C26—C25—N1—C27	0.1 (4)
C4—C3—C8—C9	179.7 (3)	C26—C25—N1—Cu1	179.3 (2)
C2—C3—C8—C9	1.5 (5)	O2—Cu1—N1—C27	−153.0 (3)
C4—C3—C8—C7	0.3 (5)	O3—Cu1—N1—C27	17.5 (3)
C2—C3—C8—C7	−177.9 (3)	N2—Cu1—N1—C27	132.6 (6)
C6—C7—C8—C9	−179.7 (3)	O2—Cu1—N1—C25	27.9 (3)
C12—C7—C8—C9	−0.7 (4)	O3—Cu1—N1—C25	−161.5 (3)
C6—C7—C8—C3	−0.2 (4)	N2—Cu1—N1—C25	−46.5 (8)
C12—C7—C8—C3	178.8 (3)	N4—C31—N2—C29	−0.6 (4)
C3—C8—C9—C10	−178.0 (3)	N4—C31—N2—Cu1	180.0 (2)
C7—C8—C9—C10	1.4 (5)	C30—C29—N2—C31	0.7 (4)
C8—C9—C10—C11	−1.3 (5)	C30—C29—N2—Cu1	−179.8 (2)
C9—C10—C11—C12	0.5 (6)	O2—Cu1—N2—C31	−12.6 (3)
C10—C11—C12—C7	0.2 (6)	O3—Cu1—N2—C31	176.8 (3)
C6—C7—C12—C11	178.9 (4)	N1—Cu1—N2—C31	61.7 (8)
C8—C7—C12—C11	−0.1 (5)	O2—Cu1—N2—C29	168.0 (3)
O4—C13—C14—C15	−55.1 (4)	O3—Cu1—N2—C29	−2.6 (3)
O3—C13—C14—C15	125.9 (3)	N1—Cu1—N2—C29	−117.6 (7)
C13—C14—C15—C16	98.1 (4)	N1—C27—N3—C26	0.2 (4)
C13—C14—C15—C20	−81.3 (4)	N1—C27—N3—C28	−179.1 (3)
C20—C15—C16—C17	1.3 (5)	C25—C26—N3—C27	−0.1 (4)
C14—C15—C16—C17	−178.1 (3)	C25—C26—N3—C28	179.2 (4)
C15—C16—C17—C18	0.4 (6)	N2—C31—N4—C30	0.2 (4)
C16—C17—C18—C19	−0.9 (6)	N2—C31—N4—C32	−177.6 (3)
C17—C18—C19—C24	−178.3 (4)	C29—C30—N4—C31	0.3 (4)
C17—C18—C19—C20	−0.2 (5)	C29—C30—N4—C32	178.0 (3)
C16—C15—C20—C21	177.5 (3)	O1—C1—O2—Cu1	2.0 (3)
C14—C15—C20—C21	−3.1 (5)	C2—C1—O2—Cu1	−179.8 (2)
C16—C15—C20—C19	−2.4 (5)	O3—Cu1—O2—C1	3.1 (6)
C14—C15—C20—C19	177.0 (3)	N1—Cu1—O2—C1	94.28 (19)
C24—C19—C20—C21	0.1 (5)	N2—Cu1—O2—C1	−94.44 (19)
C18—C19—C20—C21	−178.0 (3)	O4—C13—O3—Cu1	−1.0 (4)
C24—C19—C20—C15	−179.9 (3)	C14—C13—O3—Cu1	178.0 (2)
C18—C19—C20—C15	1.9 (5)	O2—Cu1—O3—C13	176.3 (5)
C15—C20—C21—C22	−179.8 (3)	N1—Cu1—O3—C13	85.4 (2)
C19—C20—C21—C22	0.1 (5)	N2—Cu1—O3—C13	−86.4 (2)

### Hydrogen-bond geometry (Å, °)

**Table:**

*D*—H···*A*	*D*—H	H···*A*	*D*···*A*	*D*—H···*A*
O1W—H1WA···O1	0.85	1.97	2.789 (3)	163
O1W—H1WB···O3^i^	0.85	2.07	2.904 (3)	167

Symmetry codes:  (i) −*x*+1, −*y*+2, −*z*+1.
